# A new species of terrestrially-nesting fanged frog (Anura: Dicroglossidae) from Sulawesi Island, Indonesia

**DOI:** 10.1371/journal.pone.0292598

**Published:** 2023-12-20

**Authors:** Jeffrey H. Frederick, Djoko T. Iskandar, Awal Riyanto, Amir Hamidy, Sean B. Reilly, Alexander L. Stubbs, Luke M. Bloch, Bryan Bach, Jimmy A. McGuire

**Affiliations:** 1 Museum of Vertebrate Zoology, University of California, Berkeley, CA, Berkeley, United States of America; 2 Department of Integrative Biology, University of California, Berkeley, CA, Berkeley, United States of America; 3 Department of Biology, Institute of Technology Bandung, Java, Indonesia; 4 Museum Zoologicum Bogoriense, Indonesian Institute of Sciences—LIPI, Java, Indonesia; Laboratoire de Biologie du Développement de Villefranche-sur-Mer, FRANCE

## Abstract

Herein, we describe a new species of terrestrially-nesting fanged frog from Sulawesi Island, Indonesia. Though male nest attendance and terrestrial egg deposition is known in one other Sulawesi fanged frog (*Limnonectes arathooni*), the new species exhibits a derived reproductive mode unique to the Sulawesi assemblage; male frogs guard one or more clutches of eggs festooned to leaves or mossy boulders one to two meters above small slow-moving streams, trickles, or seeps. This island endemic has thus far been collected at three sites on Sulawesi: one in the Central Core of the island, and two on the Southwest Peninsula—south of the Tempe Depression (a major biogeographical boundary). The new *Limnonectes* has the smallest adult body size among its Sulawesi congeners—with a maximum snout-vent length of about 30 millimeters. Beyond its unique reproductive behavior and body size, the species is further diagnosed on the basis of advertisement call and genetic distance from sympatric fanged frogs. The discovery and description of the new species highlights the remarkable reproductive trait diversity that characterizes the Sulawesi fanged frog assemblage despite that most species in this radiation have yet to be formally described.

## Introduction

The Asian Dicroglossid fanged frogs (genus: *Limnonectes*) include over 70 species and are stunningly complex in their reproductive biology. For example, two Malay species, *L. hascheanus* and *L. limborgi* exhibit terrestrial egg guarding by males in conjunction with nidicolous larval ontogeny: larvae hatch as free-living tadpoles yet remain in a nest guarded by the male, surviving solely on nutrients from the yolk sack [1, 2]. Four species of *Limnonectes* from Borneo, *L. kuhlii*, *L. blythii*, *L. ibanorum*, and *L. ingeri* are “voiceless”, lacking a vocal sack for advertisement calling [3]. Among them, the breeding biology of *L. blythii* includes female biased sex ratios, and males that both guard and defend limited shallow oviposition sites on gravel bars along fast-moving streams. Females of this species patrol the available nest sites to choose from the suite of deposition locations and resident male guardians [3]. In Borneo and the Philippines, both male and female *L. palavanensis* vocalize to some degree [4]. Terrestrial egg deposition is exhibited by both *L. palavanensis* and *L. parvus*; however, upon hatching, larvae are subsequently transported on the back of the male to isolated water basins in the forest [4].

The radiation of fanged frogs on the Indonesian island of Sulawesi likewise features remarkable variation in breeding biology and reproductive modes [5–8]. This poorly studied assemblage includes at least 15 species, only five of which have been formally described in the literature: *L. heinrichi* [9], *L. modestus* [10], *L. arathooni* [11], *L. microtympanum* [12], and *L. larvaepartus* [7]. Recent field investigations suggest that at least one species (referred to as *L. “Sp. I”* in [6] is voiceless, as it lacks vocal sacs and buccal slits. Brown and Iskandar [5] described terrestrial egg deposition in *L. arathooni* from Sulawesi, concurrent with other interesting observations of tadpoles spontaneously emerging from their egg capsules when nests were disturbed. Males of this species often guard multiple nests deposited on steep stream banks until newly emerged larvae wriggle down to the stream below. The reproductive modes of the Sulawesi assemblage are so varied, in fact, that they can be used as primary characters for species diagnoses. In perhaps the most striking example, the reproductive biology of the recently described *L. larvaepartus* includes internal fertilization, intraoviductal larval maturation, and the birth of free-swimming tadpoles—a first among all anurans known to science [7, 13].

**Fig 1:**
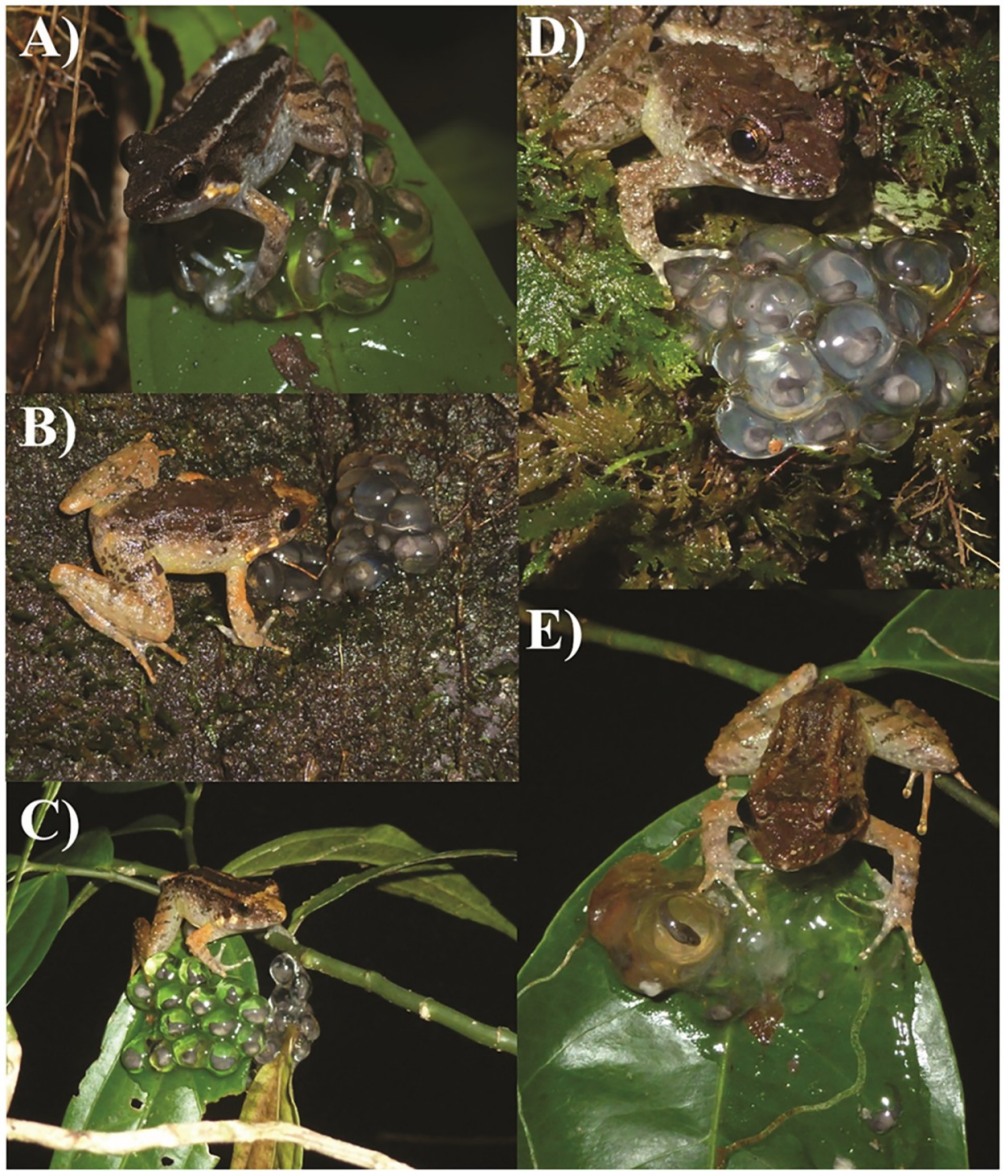
Images of *Limnonectes phyllofolia* sp. nov. in life. (A) A male *L. phyllofolia* (no voucher) guards an egg clutch on a leaf 0.2 meters above a slow spring-fed stream on Gunung Balease—24 October 2010, 21:05 h. (B) A male *L. phyllofolia*, MVZ:Herp:295234, guards an egg clutch 0.6 m up on a 2 m tall mossy boulder overhanging a stream in Bantimurung National Park—25 June 2014, 20:30 h. (C) A male *L. phyllofolia*, MVZ:Herp:295430, guards an egg clutch on a leaf 0.2 m above a puddle in Bantimurung National Park—25 June 2014, 22:25 h. (D) A male *L. phyllofolia*, MVZ:Herp:295248, guards an egg clutch on a mossy boulder 1 m above a 1 m wide cascading stream in Bantimurung National Park—25 June 2014, 21:38 h. (E) A male *L. phyllofolia* (no voucher) guards an egg clutch on a leaf while larvae hatch and drop into the water below.

Here, we report a new species of *Limnonectes* from Sulawesi, and only the second species from the island found to exhibit terrestrial egg deposition and male egg guarding. *Limnonectes* diversity on Sulawesi is poorly understood in part because of the difficulty in discriminating between morphologically and phenotypically similar animals, as well as the challenge of recognizing when morphological variation reflects interspecific differences versus intraspecific polymorphism. Our description exemplifies this challenge in that the new species occurs in sympatry with *L. arathooni*, the only other Sulawesi species documented to deposit eggs on land. Moreover, the two species are somewhat similar in size and appearance. We show herein that the new species can be diagnosed on the basis of body size, advertisement call, egg deposition behavior, and genetic distance. Unlike *L. arathooni* that deposits eggs in either streamside leaf litter or in holes in stream banks, the new species deposits nests 1–2 m off the ground on leaves or mossy boulders. These “leaf nests” overhang small, slow-moving forest streams, puddles, or seeps. As with other fanged frogs in which males exhibit parental care, we observed that males of this novel species attend one or two nests until tadpoles emerge and drop into the water below (Fig 1).

## Materials and methods

### Field sampling

**Fig 2:**
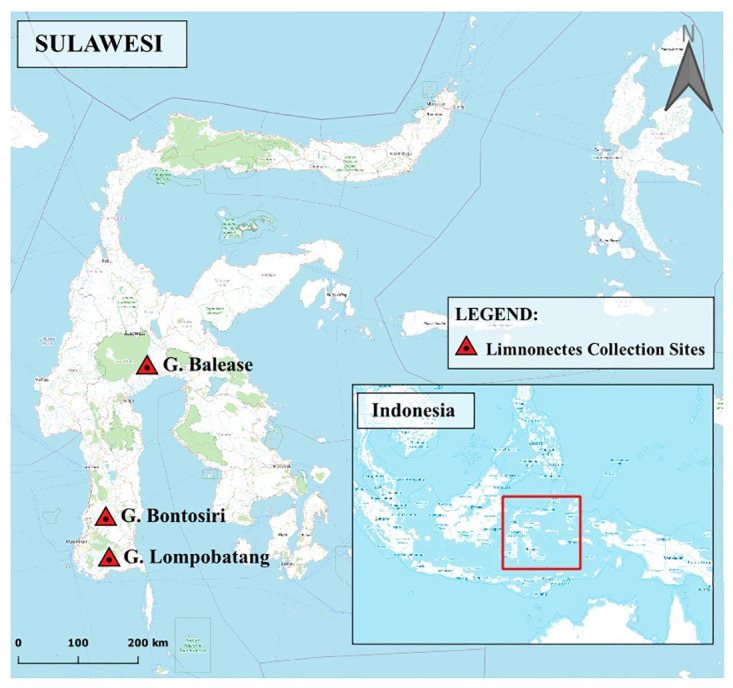
Collection localities of *Limnonectes phyllofolia* sp. nov. on Sulawesi Island, Indonesia. (A) A male *L. phyllofolia* (no voucher) guards an egg clutch on a leaf 0.2 meters above a slow spring-fed stream on Gunung Balease—24 October 2010, 21:05 h. (B) A male *L. phyllofolia*, MVZ:Herp:295234, guards an egg clutch 0.6 m up on a 2 m tall mossy boulder overhanging a stream in Bantimurung National Park—25 June 2014, 20:30 h. (C) A male *L. phyllofolia*, MVZ:Herp:295430, guards an egg clutch on a leaf 0.2 m above a puddle in Bantimurung National Park—25 June 2014, 22:25 h. (D) A male *L. phyllofolia*, MVZ:Herp:295248, guards an egg clutch on a mossy boulder 1 m above a 1 m wide cascading stream in Bantimurung National Park—25 June 2014, 21:38 h. (E) A male *L. phyllofolia* (no voucher) guards an egg clutch on a leaf while larvae hatch and drop into the water below.

Field work and animal collections were undertaken with both research and export permits in collaboration with the Indonesian Institute of Sciences (LIPI), and were granted by the Indonesian Ministry of Research, Technology, and Higher Education (RISTEK). Prior to conducting this research, animal handling and specimen preparation protocols were approved by the UC Berkeley Institutional Animal Care and Use Committee (Protocol: R279). Herpetological surveys on Sulawesi were conducted on Gunung Lompobatang in 2005, Gunung Balease in 2010, and Gunung Bontosiri (Bantimurung National Park) in 2014 (Fig 2). For downstream genomic analyses, we collected liver tissue from hand-captured frogs that were subsequently prepared and fixed using 10% buffered formalin. Animals were anesthetized and sacrificed by way of intracoelomic injection of pharmaceutical-grade benzocaine hydrochloride (250 mg/kg) diluted in 70% ethanol and buffered to a neutral pH. We injected approximately 0.03 ml for a 20g amphibian and increased or decreased the dosage accordingly based on the size of the individual specimen. The sex of each frog was determined either by viewing advertisement call behavior prior to capture, or by gonadal inspection during specimen preparation. Fixed specimens were subsequently stored in 70% ethanol and deposited at the Museum Zoologicum Bogoriense (MZB) in Cibinong, Indonesia, or the Museum of Vertebrate Zoology (MVZ) at UC Berkeley.

### Morphometrics

Morphological measurements were taken using digital calipers (to nearest 0.01 mm) including: head length (HL); head width (HW); snout-vent length (SVL); tibia length (TL); interorbital distance (IO); eye diameter (ED); internarial distance (ID); eye-nostril distance (EN); foot length (FL); tympanum diameter (TD); thigh length (THL); snout length (SL); hand length (HAL); forearm length (FLL); eye-tympanum distance (ETD), snout-nostril length (NS); upper arm length (UAL); lower arm length (LAL); and body width (BW) according to the following reference manuscript [14]. We also measured the length of the odontoid process (OPL)—the distance between the lower margin of the mandible and the top of the fang-like process protruding upward from the mandible. Notations and terminology for the digital webbing formula and relative finger lengths followed [15, 16]. In brief, fingers and toes were indicated by roman numerals, while Arabic numerals were used to indicate the position of the webbing on each phalange relative to the positions of the toe disc, intercalary cartilage, and subarticular tubercles. To validate our presumption that the new species differs morphologically from its sympatric congener (L. arathooni), we performed significance tests using a multivariate analysis of variance (MANOVA) on our morphological measurements. To account for individual- and locality-based body size variation, we first performed a principal components analysis (PCA) on the measurements taken for both species. We recorded the percent variance attributed to each PC by the morphological characters for downstream interpretation of significance testing and extracted the PC scores from each of the principal components to use as variables in the MANOVA.

### Acoustic sampling

We conducted acoustic surveys in the field, recording the advertisement call of male *Limnonectes* at a distance of 0.5–1.5 m using either a handheld solid state recorder (Marantz Professional, USA: PMD661MKII) and a stereo shotgun condenser microphone (Sennheiser: MKH 60-P48), or an iPhone attached to an external microphone. The resultant waveform audio format files were analyzed using Raven Pro Sound Analysis Software—version 1.5 [17]. For each recording, we viewed both waveform and spectrograms that were calculated using a Fast Fourier Transformation size of 512. We analyzed calls individually, thus, if recordings contained more than one call per animal, each call within the recording was isolated prior to the analysis. This demarcation resulted in a dataset consisting of 16 *L. phyllofolia* calls across 3 individuals (MVZ:Herp:295251, MVZ:Herp:295254, and MVZ:Herp:295255) and 10 *L. arathooni* calls across five individuals (MVZ:Herp:295281, MVZ:Herp:295488, MZB:Amph:33558, MZB:Amph:33559), and one non-vouchered animal). For each call, we measured number of notes, mean dominant frequency (Hz), call duration (in seconds), and pulse rate (in notes per second). We then performed cluster analysis on the call characters for both species in R—Version 3.5.1 [18]. To account for any potential statistical non-independence, multicollinearity, autocorrelation, and pseudoreplication in the data, we performed a PCA on the aforementioned call characters. We recorded the percent variance attributed to each PC by the call characters for downstream interpretation of significance tests and extracted the PC scores from the four principal components. We then used the PC scores as variables in a MANOVA to test for significant differences between the call characters of the two species.

### Genetic sampling

Field-collected liver tissue samples from *L. arathooni* (n = 21), *L. microtympanum* (n = 35), and the new species (n = 20) were preserved in RNA Later and subsequently salt extracted to obtain genomic DNA. We used the polymerase chain reaction (PCR) to amplify a 510 base pair (bp) fragment of the 16S rRNA marker with primers 16S-H3062 (5’-CCGGTTTGAACTCAGATCA-3’) and 16SB-FROG (5’-CGCCTGTTACCAAAAACAT-3’). PCR conditions were as follows: denaturation at 94°C—2 min, 35 cycles (denaturation at 94°C—45 s, annealing at 53°C—30 s, extension at 72°C—1 min), and final extension at 72°C for 1 min. Resultant amplicons were purified with ExoSAP-it (Applied Biosystems) and cycle sequenced with our amplifying primers using BigDye v 3.1. We then used ethanol (125mM EDTA) precipitation to purify the cycle sequence products and ran the samples on an ABI 3730 automated DNA sequencer (Applied Biosystems). We edited and manually aligned all sequences in Geneious 9.1.8 [19]. We then calculated uncorrected patristic distances between samples using sequences for *L. microtympanum*, *L. arathooni*, and the new species in PAUP 4.0a build 168 [20]. Sequences used in this study were uploaded to GenBank and can be accessed by referencing accession numbers: OR491124–OR491199.

### Nomeclature acts

The electronic edition of this article conforms to the requirements of the amended International Code of Zoological Nomenclature, and hence the new names contained herein are available under that Code from the electronic edition of this article. This published work and the nomenclatural acts it contains have been registered in ZooBank, the online registration system for the ICZN. The ZooBank LSIDs (Life Science Identifiers) can be resolved and the associated information viewed through any standard web browser by appending the LSID to the prefix “http://zoobank.org/”. The LSID for this publication is: urn:lsid:zoobank.org:pub:8CCB75BF-9874–472B-AC1D-2B2FDCB66905. The electronic edition of this work was published in a journal with an ISSN, and has been archived and is available from the following digital repositories: PubMed Central, LOCKSS. This species was referenced in [11] under the name *Rana palavanensis*, and as *Rana microdisca leytensis* in the British Museum of Natural History Catalogue.

## Results

*Limnonectes* ***phyllofolia*** sp. nov.

urn:lsid:zoobank.org:act:E9A254BE-026A-42A0–8A33-8CDD6D6C7715

(Figs 1 and 3–5)

**Fig 3:**
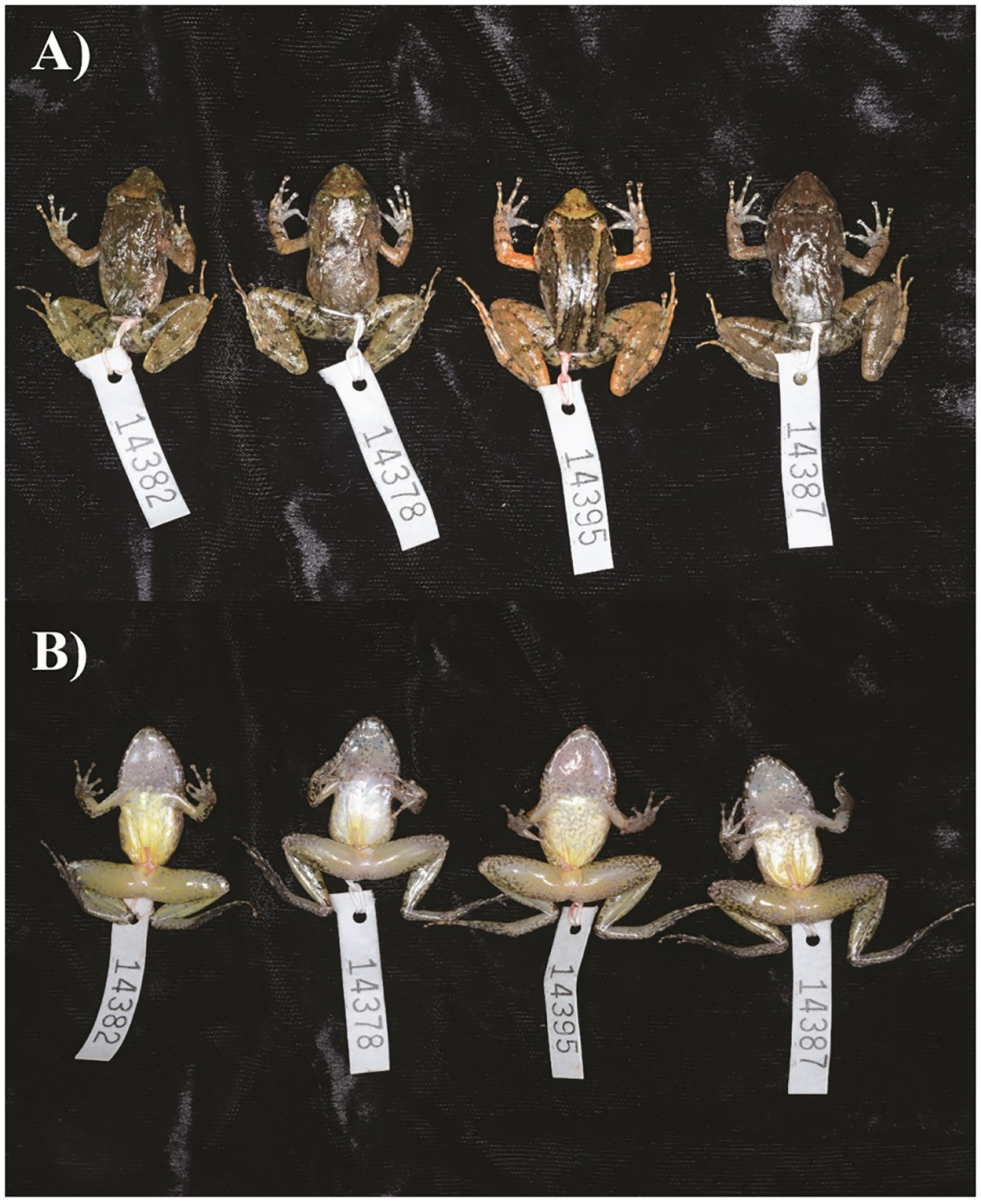
Images of a series of prepared *Limnonectes phyllofolia* showing dorsal and ventral color variation. The individuals depicted were collected on Gunung Bontosiri within Bantimurung National Park on 25 June 2014, at 592 m elevation.

**Fig 4:**
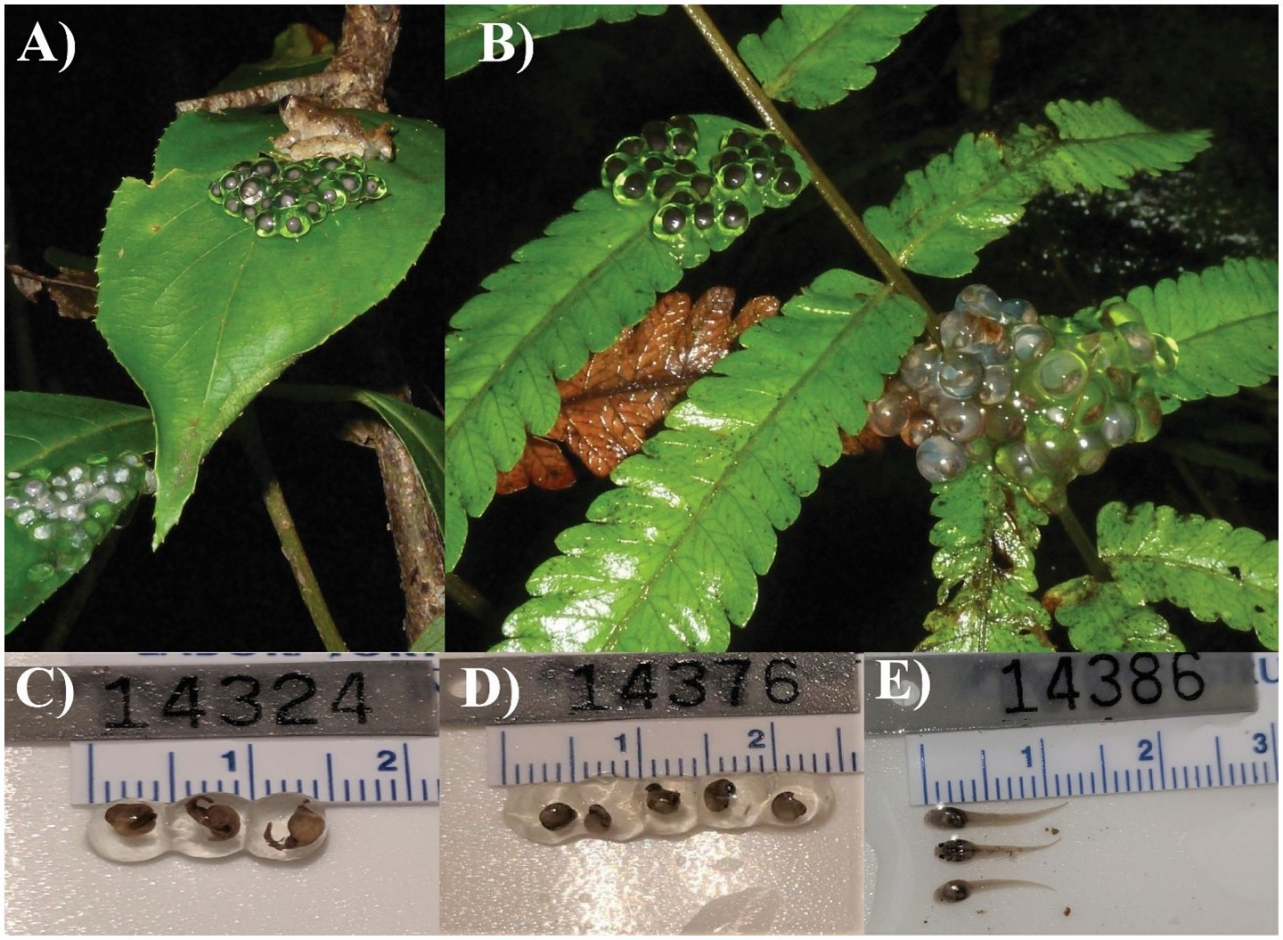
Eggs and newly hatched larvae of *Limnonectes phyllofolia*. (A) A male *L. phyllofolia*, MVZ:Herp:295236, guards two egg clutches on a sapling 2 m above a 1 m wide stream in Bantimurung National Park. (B) Example of dual egg clutches (guarded by MVZ:Herp:295224) deposited on fern frond 0.6 m above a puddle in Bantimurung National Park. (C) Example of eggs from clutch guarded by MVZ:Herp:295224—clutch was collected from leaves 0.75 m above a puddle on 24 June 2014, 19:00 h from Bantimurung National Park. (D) Example of eggs from clutch guarded by MVZ:Herp:295236—clutch was collected from leaves of a sapling tree, 2 m above a 1 m wide stream on 25 June 2014 at 21:38 h from Bantimurung National Park. (E) Example of newly hatched tadpoles. The associated clutch was guarded by MVZ:Herp:295246, and collected on a mossy boulder 1.5 m above a 1 m wide stream on 25 June 2014 at 21:38 h from Bantimurung National Park.

**Fig 5:**
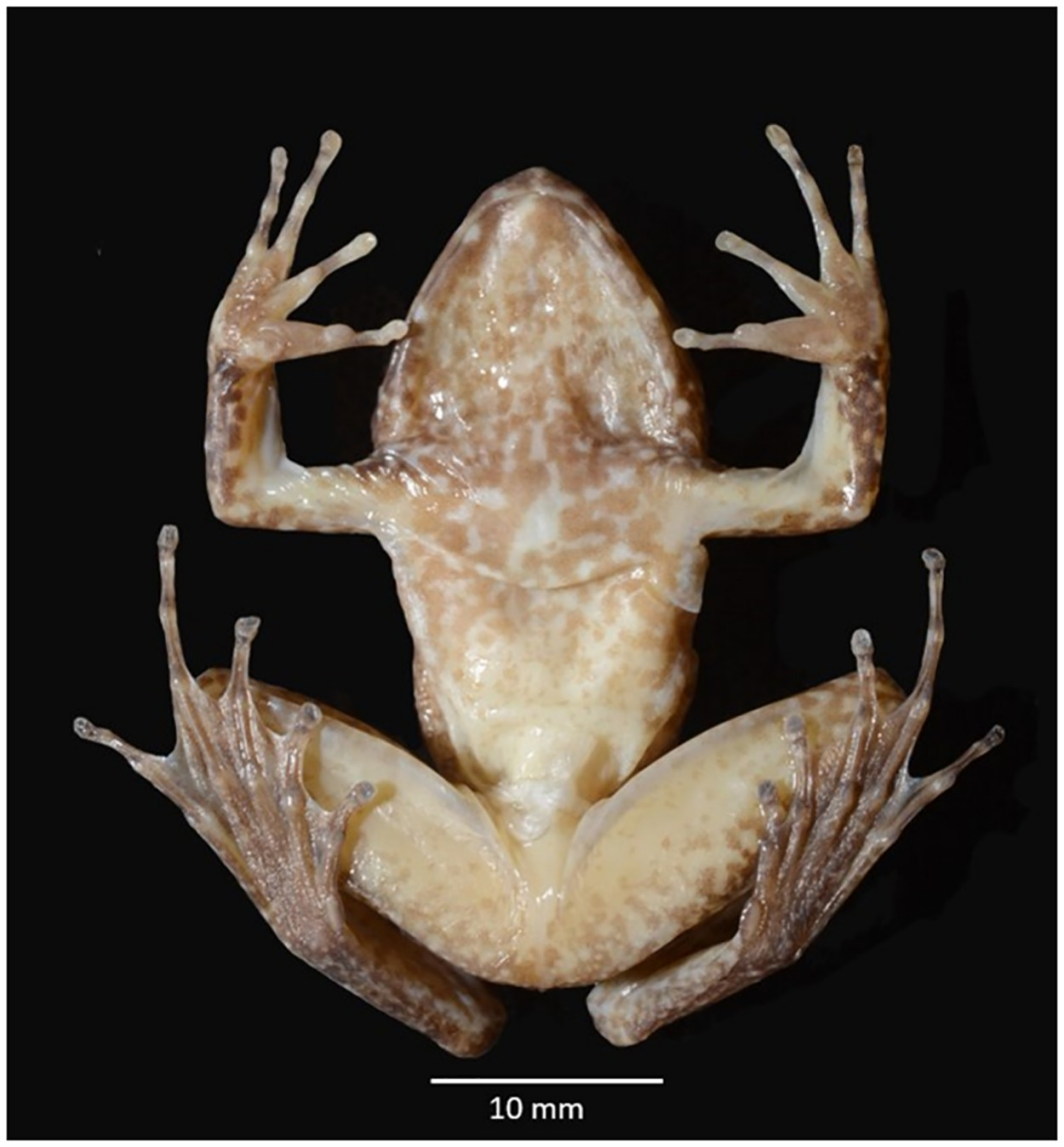
Ventral view of preserved male *L. phyllofolia* sp. nov. holotype (MVZ:Herp:295255) showing palmer and plantar aspects of the hands and feet.

### Etymology

We have informally referred to this species as *Limnonectes sp. “leaf-nester”* in reference to its characteristic reproductive mode. We therefore opted to memorialize this in its formal specific epithet, *“phyllofolia”*, which is derived from the combination of the greek *fýllo*—meaning “leaf”, and *foliá*—meaning “nest”.

### Holotype

An adult male (MZB:Amph:33560), collected 25 June 2014 at 22:08 h, from Sulawesi Island, Indonesia: Sulawesi Selatan Province: Kabupatan Maros: Kecematan Mallawa: Desa Bontosiri: Bantimurung National Park (S 04.81668, E 119.84586 ± 5 m) at 592 m by J. A. McGuire and Djoko T. Iskandar.

### Paratypes

MVZ:Herp:295232–3, MVZ:Herp:295239, MVZ:Herp:295241, MVZ:Herp:295243, MVZ:Herp:295245, and MVZ:Herp:295252, seven adult males and one adult female, all with the same data as the holotype. MVZ:Herp:295225 and MVZ:Herp:295227, two adult males; collected 24 June 2014 at 19:10 h and 21:00 h, respectively, from Sulawesi Island, Indonesia: Sulawesi Selatan Province: Kabupatan Maros: Kecematan Mallawa: Desa Bontosiri: Bantimurung National Park (S 04.79522, E 119.85471 ± 8 m) at 495 m by J. A. McGuire and Djoko T. Iskandar.

### Distribution

*Limnonectes phyllofolia* is a Sulawesi endemic, known only from the three collection localities (Fig 2) described herein (Desa Bontomaranu on Gunung Lompobatang, Gunung Balease, and Desa Bontosiri in Bantimurung National Park). Bantimurung National Park and Desa Bontomaranu are on the Southwest Peninsula of Sulawesi south of the low-lying Tempe depression, an important biogeographical boundary for many taxa including tarsiers, macaques, toads, and other fanged frog congeners [6, 21, 22]. The third locality, Gunung Balease, is located in the southeastern quadrant of Sulawesi’s Central Core, thereby demonstrating that the range of *L. phyllofolia* spans the Tempe Depression biogeographical boundary. The three collecting localities range from a low of 495 m elevation at Bontosiri to 1173 m at Bontomaranu. We expect that this species occurs broadly across the Southwest Peninsula in the lowlands up to perhaps 1200–1300 m in elevation wherever there is sufficiently intact habitat, while noting that there is desperately little intact habitat in this elevational range outside of Bantimurung National Park. In 2016, we sampled extensively on Gunung Bawakaraeng on the Southwest Peninsula between 1520 m elevation and 2800 m in mildly disturbed-to-pristine habitats and did not detect this species, suggesting that it is absent from higher elevation mossy forest. The extent of this species’ range in the Central Core is much more difficult to predict. It is possible the species is broadly distributed in intact low to mid-elevation habitats, but pristine forest is rare in the lowlands of the Central Core and where we have surveyed such habitats (e.g., within Lore Lindu National Park), we did not encounter this species. It is possible that this species was once widespread in lowland forests of the Central Core but is now range-restricted because of loss of habitats at lower elevations.

### Diagnosis

*L. phyllofolia* sp. nov. can be diagnosed on the basis of the following combination of character states: (1) small body size, (2) reduced webbing, (3) unique advertisement call, (4) a heretofore unique reproductive mode, and (5) by geographic range—being restricted to localities on the Southwest Peninsula and the southeastern quadrant of Sulawesi’s Central Core.

### Comparisons

We have found *Limnonectes phyllofolia* living in sympatry/syntopy with the described species *L. arathooni* (at Bontomaranu) and *L. microtympanum* (at Bontomaranu and Bontosiri). With regard to the informally recognized undescribed species mentioned by our collegues in [6, 8], we have found *L. phyllofolia* in sympatry with the undescribed *L. “sp. 2”* (at Gunung Balease), with the undescribed *L. “sp. T”* (at Gunung Balease), and with the undescribed *L. “sp. J”* (at Gunung Balease). The range of *L. phyllofolia* likely also overlaps with the ranges of *L. “sp. D”*, *L. “sp. G2”*, and *L. “sp. I”*. The known geographic distribution of *L. phyllofolia* does not overlap with the ranges of *L. modestus*, *L. heinrichi*, *L. “sp. 1”*, or *L. “sp. J2”*. Regardless of whether there is range overlap, *L. phyllofolia* is highly genetically distinct from each of these congeners.

*Limnonectes phyllofolia* is distinguished morphologically from all other described Sulawesi *Limnonectes* by the following combination of characters: small adult size (a maximum SVL of 30 mm, Table 1), highly reduced webbing, and the presence of a post-orbital skin groove which appears as an off-white stripe on lighter-colored individuals (though reduced webbing and the postorbital groove have also been reported in *L. arathooni* [6]. *Limnonectes phyllofolia* can also apparently be distinguished from all other Sulawesi *Limnonectes* on the basis of its reproductive behavior (though we admittedly still don’t know the reproductive modes of several species). Namely, this species is terrestrially-nesting, depositing its masses in tightly packed clutches of 10–20 eggs on leaves or boulders festooned with a thick layer of wet moss immediately over- or adjacent to small streams and seeps. Though nest attendance is also known in *L. arathooni*, an only slightly larger congener; nest sites of *L. arathooni* occur on steep stream banks, and larvae hatch from eggs when disturbed, sliding or wriggling down the bank into water [6]. In contrast, sites of *L. phyllofolia* occur 1–2 m off the ground, either on leaves of ferns, saplings, or other plants that overhang small slow-moving streams, seeps, or puddles, or on elevated boulders overhanging water (Figs 1 and 4). It is unclear whether the new species overlaps in range with the recently described *L. larvaepartus*, a small species that is nevertheless substantially larger in adult body size, with mean male and female SVL of *L. larvaepartus* being reported as 37.4 mm and 40.2 mm, respectively [7]. As *L. larvaepartus* demonstrates a reproductive mode unique to all frogs (internal fertilization with live birth of tadpoles), the new terrestrially-laying species can be easily distinguished from its congener by this criterion [7]. Though morphological measurements suggest that *L. arathooni* can be distinguished from *L. phyllofolia* by their larger size (*L. arathooni* average SVL = 35.23 mm, range: 29.47–44.3 mm; *L. phyllofolia* average SVL = 27.03 mm, range: 21.53–30.13; S1 Table), field identification between of the two species may be difficult from size alone. Thus, *L. phyllofolia* can also be distinguished from *L. arathooni* and all other *Limnonectes* on the basis of call: a rapid series of clicks unlike any of its Sulawesi congeners.

**Table 1:** Range of morphological character measurements (in mm) of adult *L. phyllofolia* sp. nov. type specimens.

*Limnonectes phyllofolia Morphological Characters*							
	Males (27)		Females (2)		Juveniles (6)		Holotype
Head length (HL)	6.24–10.60	**(9.24)**	9.27–10.04	**(9.66)**	5.99–7.89	**(6.93)**	9.4
Head width (HW)	8.70–12.54	**(10.34)**	10.49–10.8	**(10.65)**	6.95–8.41	**(7.56)**	11.38
Snout-Vent Length (SVL)	21.53–30.13	**(27.03)**	26.70–29.29	**(28.00)**	18.13–22.23	**(19.67)**	28.12
Tibia Length (TL)	12.55–16.38	**(14.82)**	13.99–16.80	**(15.40)**	9.56–12.07	**(10.96)**	16.18
Interorbital Distance (IO)	1.91–3.12	**(2.67)**	2.56–2.86	**(2.71)**	1.94–2.47	**(2.18)**	2.98
Eye Diameter (ED)	3.04–4.25	**(3.50)**	3.01–4.00	**(3.51)**	2.71–3.08	**(2.82)**	4.14
Internarial Distance (IN)	2.05–2.89	**(2.50)**	2.38–2.55	**(2.47)**	1.70–2.29	**(1.92)**	2.58
Eye-Nostril Distance (EN)	1.94–2.96	**(2.44)**	2.58–2.71	**(2.65)**	1.66–2.25	**(1.98)**	2.58
Foot Length (FL)	12.07–16.41	**(14.25)**	12.08–15.85	**(13.97)**	4.70–11.96	**(9.42)**	15.48
Tympanum Diameter (TD)	1.34–2.82	**(2.12)**	2.25–2.30	**(2.28)**	1.28–2.13	**(1.62)**	2.56
Thigh Length (THL)	12.06–16.37	**(14.24)**	13.71–15.47	**(14.59)**	9.31–11.39	**(10.48)**	16.37
Snout Length (SL)	2.12–4.15	**(3.37)**	3.57–3.72	**(3.65)**	2.30–3.08	**(2.76)**	3.9
Hand Length (HAL)	6.13–8.81	**(7.59)**	7.79–8.35	**(8.07)**	4.41–5.80	**(5.21)**	8.15
Forearm Length (FLL)	4.38–6.28	**(5.34)**	4.98–6.43	**(5.71)**	3.00–4.60	**(3.97)**	5.25
Eye-Tympanum Distance (ETD)	0.62–1.54	**(1.03)**	1.02–1.09	**(1.06)**	0.52–0.72	**(0.64)**	1.21
Snout-Nostril Length (NS)	0.72–1.32	**(1.01)**	0.94–1.01	**(0.98)**	0.74–1.10	**(0.92)**	1
Upper Arm Length (UAL)	4.69–6.80	**(5.59)**	5.28–6.20	**(5.74)**	3.67–4.69	**(4.18)**	5.26
Lower Arm Length (LAL)	10.54–14.41	**(12.85)**	12.53–13.67	**(13.1)**	8.29–10.14	**(9.35)**	13.99
Body Width (BW)	7.14–11.11	**(9.13)**	8.24–8.99	**(8.62)**	5.79–7.59	**(6.68)**	9.78
Odontoid Process Length (OPL)	0.70–1.32	**(0.97)**	0.77–1.01	**(0.89)**	0.45–0.72	**(0.62)**	1.14
HL/SVL		**(0.34)**		**(0.34)**		**(0.35)**	0.33
HW/SVL		**(0.38)**		**(0.38)**		**(0.38)**	0.40
SL/SVL		**(0.12)**		**(0.13)**		**(0.14)**	0.14
EN/SVL		**(0.09)**		**(0.09)**		**(0.10)**	0.09
IN/SLV		**(0.09)**		**(0.09)**		**(0.10)**	0.09
ETD/SVL		**(0.04)**		**(0.04)**		**(0.03)**	0.04
OPL/SVL		**(0.04)**		**(0.03)**		**(0.03)**	0.04
TD/SVL		**(0.08)**	.	**(0.08)**		**(0.08)**	0.09
IO/SVL		**(0.10)**		**(0.10)**		**(0.11)**	0.11
ED/SVL		**(0.13)**		**(0.13)**		**(0.14)**	0.15
TL/SVL		**(0.55)**		**(0.55)**		**(0.55)**	0.58
THL/SVL		**(0.53)**		**(0.52)**		**(0.53)**	0.58
FL/SVL		**(0.53)**		**(0.50)**		**(0.48)**	0.55
HW/HL		**(1.12)**		**(1.10)**		**(1.10)**	1.21
SL/HW		**(0.33)**		**(0.34)**		**(0.36)**	0.34
IO/IN		**(1.07)**		**(1.10)**		**(1.15)**	0.86

^1^ Averages are given in parentheses for morphological character measurements (in mm), including SVL corrected measurements (in mm).

### Description of holotype

Adult male, SVL 28.12 mm, head large and wide relative to body size: HL 9.4 mm, HW 11.38, 33% and 40% of SVL, respectively; head slightly wider than long, 115% of HL, and wide relative to the body (HW/BW = 116%); internarial distance (2.58 mm) roughly equal to the distance from the nostril to the anterior margin of the eye (though in paratypes the latter may be slightly longer (mean IN/EN = 102%)); eye large, 4.14 mm in diameter, 15% of SVL, and 44% of HL; interorbital distance 2.98 mm, slightly convex, and 11% of SVL; tympanum round, 2.56 mm in diameter, 9% of SVL; odontoid processes (fang-like boney projections protruding upward from the mandible) small, 1.14 mm.

There is a distinct supra-tympanal fold of skin initiating at the center posterior margin of the eye that extends over and around the tympanum, terminating just above the arm.

Arms stocky and short, with forearm length (FAL = 5.25 mm) roughly equal to upper arm length (UAL = 5.26 mm), and both distances 64.4% and 64.5% of hand length (HL = 8.15 mm), respectively; fingers long, with hand length 29% of SVL and 86% the length of the head; relative finger lengths III > I > II > IV with webbing completely absent from fingers (Fig 5); body squat, a third wide as it is long (BW/SVL = 34%); hindlimbs long and slender, with tibia length roughly equal to thigh length (TL / THL = 98.8%), both distances only slightly longer than the foot (FL = 15.48 mm); hindfoot webbing highly reduced (relative to sympatric *Limnonectes* spp.) with webbing formula I 11/2—2- II 1—1 III 3-—1 IV 2-—3 V (Fig 5); skin fairly rugose, especially behind the eyes, but also dorsally and laterally along the flanks; skin smooth on the ventral side of the limbs and trunk.

### Coloration

Prominent markings (even in preservative) include: a small (< 1 mm), perfectly circular light-colored spot in the center of the snout, a light-colored interorbital bar, a light-colored delta-shaped snout patch initiating between the eyes and terminating at the snout, and vertical white lip bars alternating across the length of the mandible and terminating beneath the tympanum, eyes, nares, and snout, respectively. In individuals without a light-colored snout patch, the interorbital bar is dark and may also present with a distinct groove in the dermis (Fig 3). Dark leg bars are usually prominent, especially on the thigh, and appear to run continuously across the thigh, tibia, and tarsus when the legs are not extended. Often, the leg bars will be faded or absent across the tibia. In life, *L. phyllofolia* coloration ranges from very dark, to light cinnamon brown (Fig 2). Two color morphs are generally exhibited: standard, and barred. Standard morphs are dorsally brown, usually grading to lighter brown or cream-colored flanks (e.g., Figs 1B, 1D and 3A [MVZ:Herp:295255, 295239, 295248]). Barred individuals have two light-colored stripes that initiate postorbitally and run the length of the dorsum on either side of the spine and urostyle, terminating at the vent (e.g., Figs 1A, 1C, 1E and 3A [MVZ:Herp:295256]). Gray-brown mottling is prominent on the cream-colored venter, initiating at the snout and extending down the trunk. The same mottling is also present around the ventral margins of the legs, where the cream-colored background may in some cases grade to a pale yellow color.

### Eggs and tadpoles

Average *Limnonectes phyllofolia* sp. nov. clutch size of n = 9 nests collected in concert with adult male specimens was 15 (range = 10–21 eggs) (Fig 4). The roughly 5 mm diameter eggs are sturdy, appear perfectly spherical, and are tightly packed within the mound-like nest. Embryos are surrounded by clear jelly, such that developing larvae are easily visible. In the field, we observed that both late-stage and non-viable eggs become cloudy, and are likely susceptible to mold and other fungi when not accompanied by a guardian male.

### Natural history

Of the three known localities where *Limnonectes phyllofolia* has been found, one was in pristine karst forest habitat within Bantimurung National Park, one was in mature regenerated forest adjacent to primary forest on Gunung Balease, and one was adjacent to a large, high-flow waterfall in a heavily disturbed village setting (Desa Bontomaranu on Gunung Lompobatang). Despite having observed these frogs in habitats of variable quality, the fact that we have not found this species at the dozens of additional (mostly disturbed) sites that we have surveyed throughout the Southwest Peninsula and southern Central Core suggests to us that this species is likely almost entirely restricted to mature natural forest habitats below 1100 meters in elevation. It is quite possible that this lowland obligate, and the sampling site at the base of G. Lompobatang (1100 m elevation) may itself be an elevational outlier. Mature lowland forest is sufficiently scarce on Sulawesi to the extent that any species restricted to these habitats will be found primarily in protected areas such as Bantimurung National Park, or in remote sites away from human habitations that retain some suitably natural forest vegetation (e.g., our 700–900 m elevation collecting sites on Gunung Balease).

*Limnonectes phyllofolia* is one of several small Sulawesi fanged frog species that are fairly terrestrial in their habits. Although reproduction is associated with small streams and seeps, individuals can be found far from any free-flowing water in open forest leaf-litter. We collected several individuals on Gunung Balease that were hundreds of meters from the one breeding site that we identified. That breeding site was a spring-fed seep that emerged from the forest floor, flowed slowly for perhaps 50 meters down slope, and then disappeared. We were not aware of a more continuous stream within 1.5 km of this spring-fed seepage system. Small discontinuous aquatic habitats are likely to be colonized by smaller more terrestrial *Limnonectes* species such as *L. phyllofolia*, and these habitats are unlikely to be utilized by larger, more stream-adapted *Limnonectes* species that might be both predators on-, and competitors with this species.

At both Gunung Balease and Gunung Bontosiri, we found many males perched on egg clutches immediately over small, slow-moving streams. At Bontosiri, we also found a small number of males on clutches adjacent to a moderately large stream (4–5 m in width) with a faster flow regime, as well as on the margin of a large pool (12 m x 6 m) nearly devoid of flow. The egg clutches were either on the green leaves of tree saplings or ensconced in moss on boulders. We only saw males attending egg clutches at night and unattended egg clutches were observed on Gunung Balease during the day. Based on the close proximity of some clutches (6–12 inches apart), it appeared that some individual males might be guarding two clutches at once, although we never confirmed this through observation of a male switching between clutches. Although we observed many males on egg clutches, none were vocalizing. Three males were observed calling and none were on or near egg clutches. Having not witnessed amplexus, it remains unclear which sex chooses the site for egg deposition, but it seems possible that females choose males in part based on their territory quality. It is also possible, however, that the females decide where the clutches will be deposited once engaged in amplexus.

### Variation

In addition to variations in coloration described above, this species also varies substantially in degree of rugosity of the dorsal skin surfaces, with some individuals quite rough in appearance and other smooth. Morphometric variation among the paratypes and holotype are described in Table 1. Averages are given in parentheses for morphological character measurements (in mm), including SVL corrected measurements (in mm).

### Morphometrics

**Fig 6:**
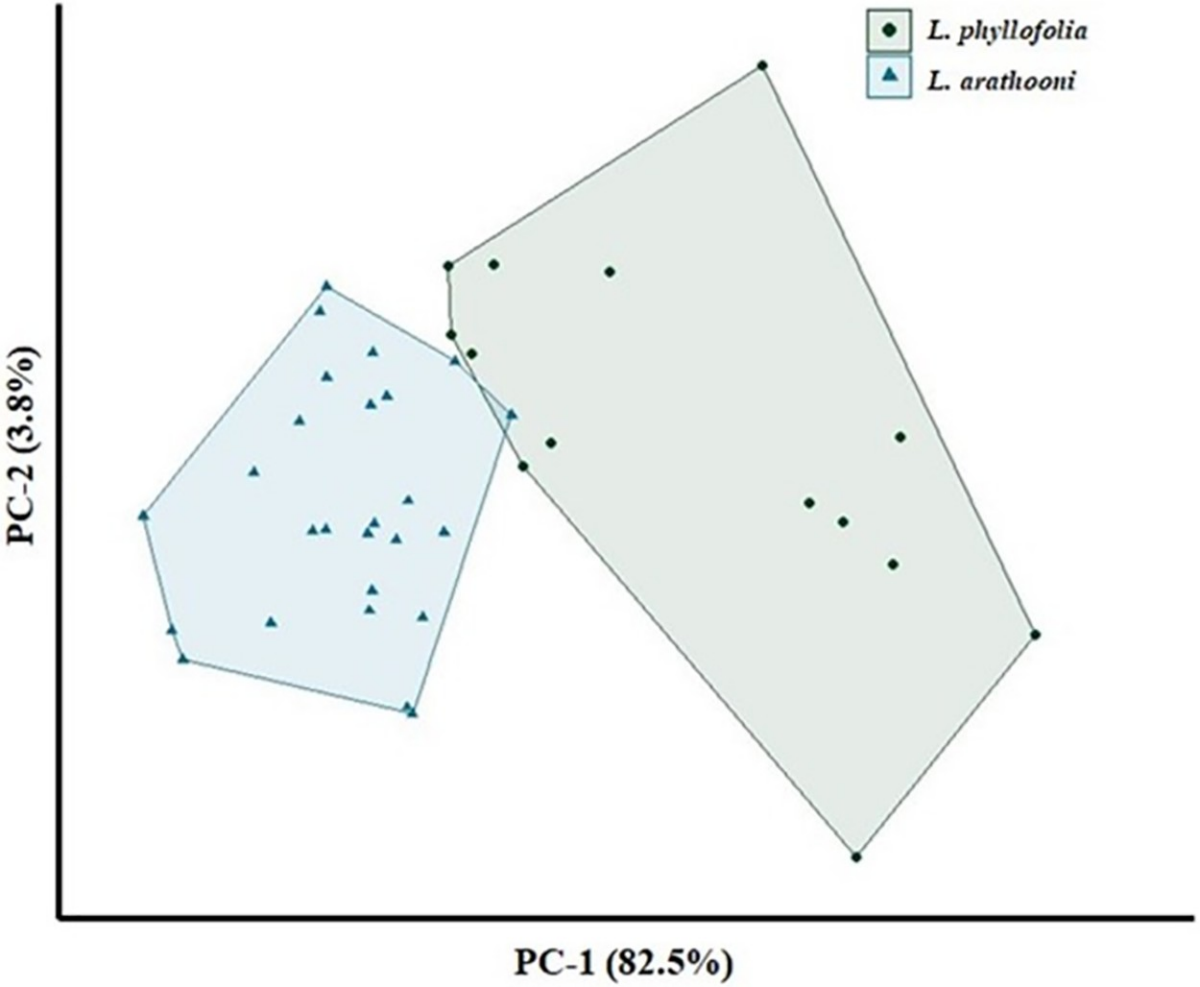
Results of principal components analysis on morphological characters. Biplot shows clustering of PC scores from morphological measurements by species. In PC-1, over 55% of the variance contribution within the model is attributed to the opposition (5.5% each) of ten morphological characters: THL, LAL, HW, SVL, TL, FL, HAL, FLL, BW, and UAL. In PC-2, 88% of the variance contribution within the model is attributed to the opposition of five morphological characters: ED, TD, SN, ETD, SL.

Though *L. phyllofolia* is objectively smaller than *L. arathooni*, it remains possible that observers might confuse the two species in the field. Ergo, we report statistical support for the new species being distinguishable from its sympatric congener based upon body size and limb lengths (Fig 6). Eigenvalues from the cluster analysis on morphological characters revealed that the first three principal components (PCs) accounted for 89.4% of the total variance across the morphological dataset. Important contributors of variance to the first three PCs were: (1) THL, LAL, HW, SVL, TL, FL, HAL, FLL, BW, and UAL for PC-1, (2) ED, TD, SN, ETD, and SL for PC-2, and (3) TD, ED, SN, OPL, EN, and ETD for PC-3. MANOVA results on the PC scores were highly significant, highlighting the substantial differences between the morphological characters of the two species (*α* < 0.001; P = 9.38e-07). The above ten characters in PC-1 explained most of the total variance (82.47%) in the model and PCA scores in this dimension differed significantly (*α* < 0.001; P = 5.408e-09).

### Advertisement call

**Fig 7:**
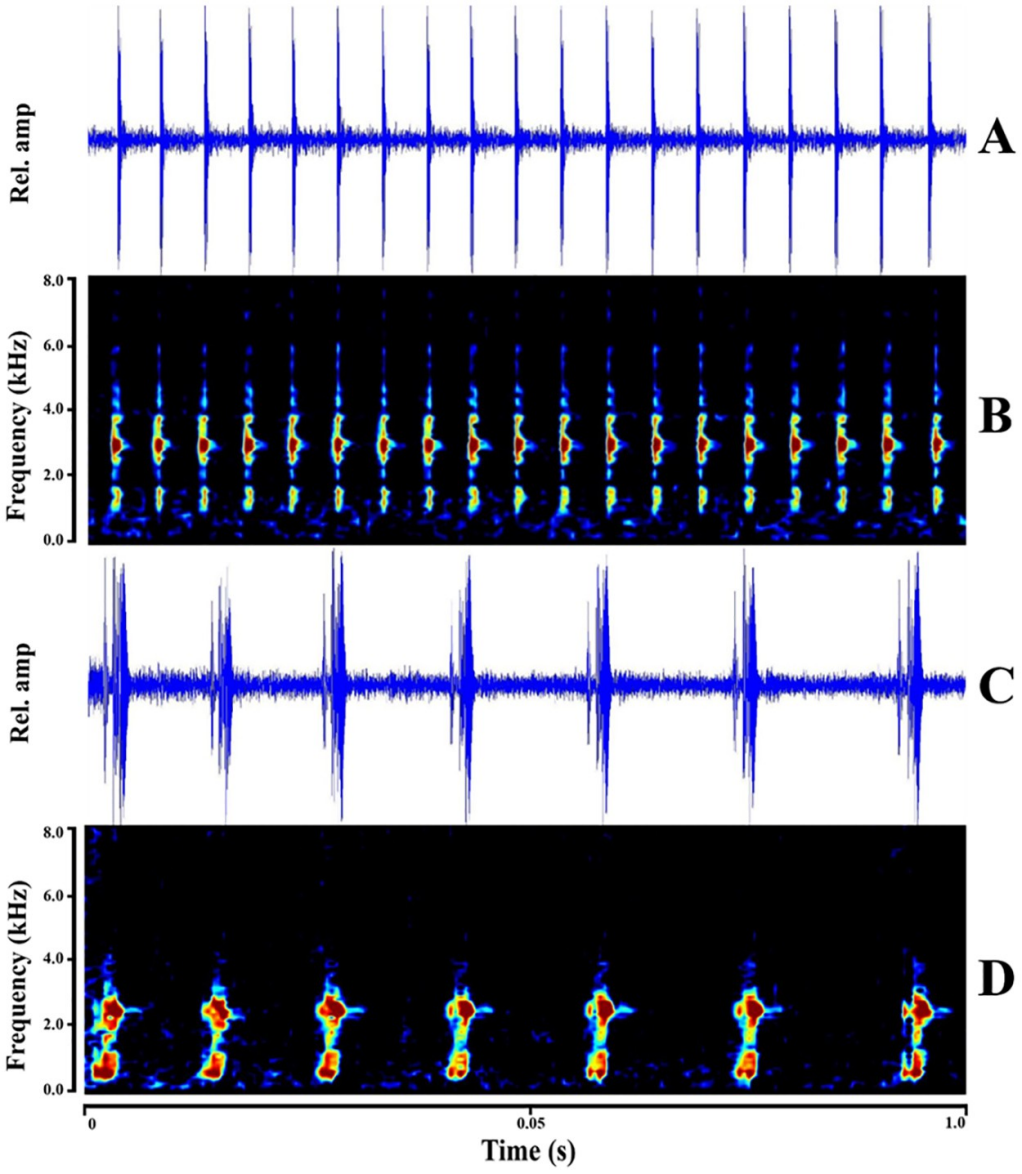
Results of principal components analysis on morphological characters. (A) Waveform oscillogram (relative amplitude vs. time in seconds) and corresponding (B) spectrogram (kilohertz vs. time in seconds) of a 19 note advertisement call of *L. phyllofolia* sp. nov. (MVZ:Herp:295251) at Gunung Balease. The one second call was recorded from an approximate distance of one meter by J. A. McGuire on 27 June 2014, 23:30 h. (C) Waveform oscillogram and corresponding spectrogram (D) of a representative 7 note advertisement call of *L. arathooni* (MVZ:Herp:295281), calling from a mossy boulder, 1 m above ground level, above a 1 m wide stream in Bantimurung National Park. The one second call was recorded from an approximate distance of 1 meter by J. A. McGuire on 25 June 2014, 22:02 h.

Here, we compare the advertisement call of *L. phyllofolia* with its most similar Sulawesi congener, *L. arathooni*—a species with which it is also sympatric on Sulawesi’s Southwest Peninsula. Indeed, *L. phyllofolia* can be easily distinguished from *L. arathooni* (and all other Sulawesi congeners) on the basis of call—a rapid series of clicks quite unlike the high-pitched chirps uttered by *L. arathooni*. Examples of calls from both species are shown in Fig 7. Call duration of *L. phyllofolia* ranged from 0.596–2.86 seconds (sec) ($\bar{x}=1.3$ sec, SD = 0.86), while the range of call duration recorded for *L. arathooni* was 0.355–3.537 sec ($\bar{x}=1.69$ sec, SD = 1.19). Number of notes from calls of *L. phyllofolia* ranged from 11–54 notes ($\bar{x}=32.25$ notes, SD = 16.38), while *L. arathooni* calls were composed of substantially fewer notes (range = 2–7 notes, $\bar{x}=3.4$ notes, SD = 1.71). The pulse rate of calls collected from *L. phyllofolia* varied little, ranging from 16.78–19.44 notes per second (NPS) ($\bar{x}=18.34$ NPS, SD = 0.62), while the pulse rate of calls from *L. arathooni* ranged from 0.57–6.13 NPS ($\bar{x}=3.16$ NPS, SD = 2.54). Dominant frequency of *L. phyllofolia* ranged from 1142.70–2818.89 Hz ($\bar{x}=2046.89$ Hz, SD = 536.81), while dominant frequency of across *L. arathooni* calls ranged from 1751.40–2813.67 Hz ($\bar{x}=2374.55$ Hz, SD = 312.09).

**Fig 8:**
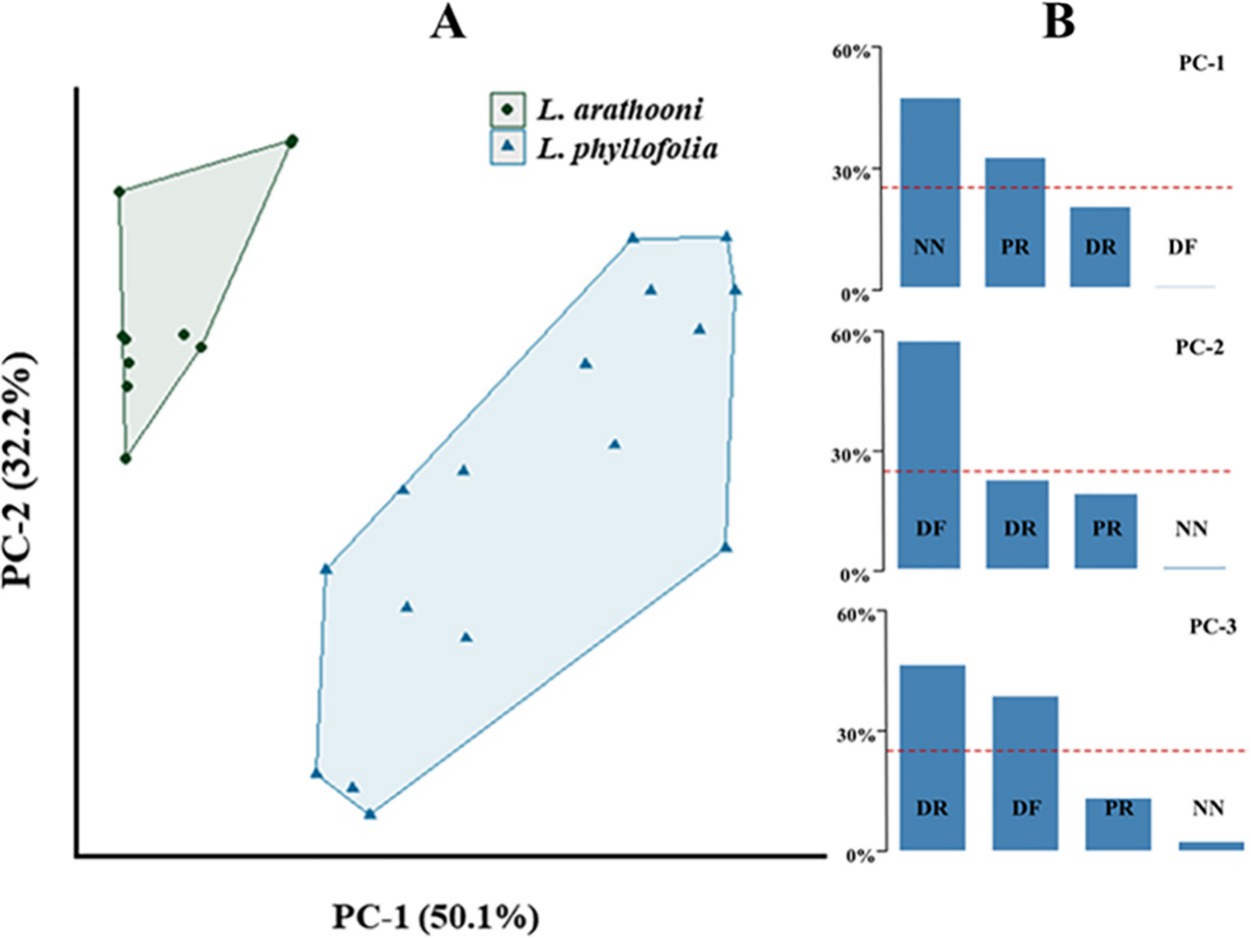


Cluster analysis with PCA resulted in the first three of four PCs accounting for 98.38% of the total variance across the call character dataset (Fig 8A). Important contributors of variance to the first three PCs were note number and pulse rate for PC1, dominant frequency for PC-2, and call duration and dominant frequency for PC-3 (Fig 8B). MANOVA results on the PC scores were highly significant, highlighting substantial differences between call characters of the two species (*α* < 0.001; P = 2.383e-16). For PC-1, which was explained by the variances of pulse rate and note number, score differences were significant between the two species at *α* < 0.001 (P = 4.973e-07). PC-2 was explained mostly by the variance contributed by dominant frequency, and scores again differed significantly between the two species at *α* = 0.001 (P = 9.592e-03). There were no significant differences between PC scores of PC-3 and PC-4. Call differences between *L. phyllofolia* and *L. arathooni* are quite clear, both audibly and via visual inspection of sonograms (Fig 7), though here we offer statistical validation that *L. phyllofolia* can be diagnosed from its similarly-sized sympatric congener on the basis of note number, pulse rate, and dominant frequency. The resulting biplot from the PCA (Fig 8A) shows clustering in the data by species as well as the variance contribution by call characters for the first three PCs.

### Genetic distance

Using data collected for another project, we calculated uncorrected patristic distances for a 510 bp fragment of the 16S mitochondrial gene for *L. arathooni*, *L. microtympanum*, and *L. phyllofolia*. We found that the currently recognized species L. arathooni and L. microtympanum exhibited a minimum uncorrected pairwise genetic distance of 3.85% from one another, whereas these species exhibited a minimum of 6.7% and 6.9% uncorrected genetic distances from *L. phyllofolia*, respectively. Notably, all three species were collected together at one site (Bontomaranu), and *L. phyllofolia* was collected in sympatry with *L. microtympanum* at a second site (Bontosiri).

## Discussion

The poorly studied radiation of Sulawesi fanged frogs likely represents the most diverse amphibian assemblage on the island. Five species are formally described, yet recent studies, ongoing field surveys, and genetic analyses indicate that the assemblage is comprised of at least 15 species [6–8]. Moreover, this group has been suggested to represent an adaptive radiation [8]. For example, Sulawesi *Limnonectes* exhibit at least 450-fold variation in adult body mass, important differences in microhabitat use and associated eco-morphological phenotypes, a diverse array of reproductive modes, and a large number of species that can be found in sympatry (we have found at least 6 species co-occurring in the Central Core and on the Southeast Peninsula, and the known extents of species geographic ranges leave possible that as many as 11 species might be found together at select localities in the eastern Central Core). A suite of sympatric ecomorphs can reliably be found in lowland and montane habitats across the island. For instance, several large and moderately-large fully-webbed species (*L. heinrichi*, *L. microtympanum*, *L. “sp. D”*, *L. “sp. I”*, *L. “sp. 2”*) are strongly associated with large, fast moving streams. Smaller extensively-webbed species (such as *L. modestus* and *L. larvaepartus* are sometimes found on large streams but are more typically associated with smaller streams and seeps. More terrestrial leaf-litter specialists such as *L. arathooni*, *L. “sp. G2”*, *L. “sp. J”*, *L. “sp. J2”*, *L. “sp. T”*, and *L. “sp. 1”* have much reduced webbing even though most utilize small streams for reproduction. *Limnonectes phyllofolia* is an example of this latter terrestrial ecomorph: it is the smallest known Sulawesi fanged frog, it has greatly reduced interdigital webbing, and we have collected the species quite far from any source of surface-water. This species, like the other more terrestrial species, appears to avoid larger streams and rivers, instead breeding adjacent to small streams and seeps where it exhibits a reproductive mode wherein eggs are deposited on leaves or in thick, moist moss overhanging water. There remains much to be done in the way of identifying and characterizing ecomorphological variation in Sulawesi fanged frogs, but the discovery and description of *L. phyllofolia* provides further evidence that *Limnonectes* frogs partition habitats and niches on the island of Sulawesi.

At this time, there is still considerable uncertainty regarding the full geographic distribution of *L. phyllofolia*, particularly regarding its range within Sulawesi’s Central Core. We have failed to find additional populations despite having conducted extensive fieldwork in the southern half of the Central Core where *L. phyllofolia* should be expected. However, the Central Core is primarily composed of an upland plateau and it’s possible that this species may be (or may have once been) restricted to the lower elevation margins of the main massif with anthropogenic habitat modification resulting in extirpation of the species over much of its range north of the Tempe Depression. Further survey work is clearly required to fully assess the geographic extent of this species range, and it is essential to locate and investigate lowland regions in the Central Core that retain intact forest habitats.

Indonesia has been repeatedly identified as a global biodiversity hotspot [23–25], and Sulawesi is itself a global hotspot of biodiversity and endemism, and a high-priority, imperiled region of conservation concern [25–28]. Nevertheless, the herpetofauna of this island remains poorly documented with many of the currently recognized species actually representing species complexes, and numerous additional morphologically distinct species awaiting taxonomic description [29]. Here, we add an additional species to the known roster of anurans inhabiting Sulawesi, while noting that much work remains to fully characterize the herpetofauna of this remarkable island.

## Supporting information

S1 Table Limnonectes arathooni. Range of body size and limb length measurements (in mm) of n = 14 adult *Limnonectes arathooni* specimens used in our comparative morphological analyses (averages given in parentheses).
(PDF)

S1 File Specimen Catalog IDs, morphological data, and acoustic data used in this study. (XLSX)

S2 File (PDF)
