# Supplementary material for: A new species of terrestrially-nesting fanged frog (Anura: Dicroglossidae) from Sulawesi Island, Indonesia
Source: PLoS One. 2023 Dec 20;18(12):e0292598. doi: 10.1371/journal.pone.0292598 (PMC10732399; doi:10.1371/journal.pone.0292598)
Supplement: S1 Table — Range of body size and limb length measurements (in mm) of n = 14 adult Limnonectes arathooni specimens used in our comparative morphological analyses (averages given in parentheses). (PDF) [file pone.0292598.s001.pdf]

S1 Table. Range of body size and limb length measurements (in mm) of *n*=14 adult *Limnonectes arathooni* specimens used in our comparative morphological analyses (average given in parentheses).

Limnonectes arathooni		
	Adults (14)	
Head length (HL)	9.91—14.51	(12.18)
Head width (HW)	12.86—17.54	(14.55)
Snout-Vent Length (SVL)	29.47—44.30	(35.23)
Tibia Length (TL)	14.90—24.35	(19.89)
Interorbital Distance (IO)	2.44—4.58	(3.46)
Eye Diameter (ED)	2.75—5.22	(3.85)
Internarial Distance (IN)	2.85—4.52	(3.55)
Eye-Nostril Distance (EN)	2.25—3.55	(2.88)
Foot Length (FL)	14.66—23.06	(19.15)
Tympanum Diameter (TD)	2.13—2.87	(2.54)
Thigh Length (THL)	14.81—24.44	(19.59)
Snout Length (SL)	2.12—4.15	(4.75)
Hand Length (HAL)	7.84—11.79	(9.99)
Forearm Length (FLL)	5.50—9.15	(6.96)
Eye-Tympanum Distance (ETD)	1.21—2.10	(1.58)
Snout-Nostril Length (NS)	0.97—1.90	(1.43)
Upper Arm Length (UAL)	5.90—8.72	(7.09)
Lower Arm Length (LAL)	13.70—19.00	(16.4)
Body Width (BW)	8.77—18.88	(12.73)
Odontoid Process Length (OPL)	0.98—2.08	(1.45)
