# Supplementary material for: A new species of terrestrially-nesting fanged frog (Anura: Dicroglossidae) from Sulawesi Island, Indonesia
Source: PLoS One. 2023 Dec 20;18(12):e0292598. doi: 10.1371/journal.pone.0292598 (PMC10732399; doi:10.1371/journal.pone.0292598)
Supplement: S2 File — (PDF) [file pone.0292598.s003.pdf]

Copyright and License

OpenStreetMap® is *open data*, licensed under the Open Data Commons Open Database License (ODbL) by the OpenStreetMap Foundation (OSMF).

You are free to copy, distribute, transmit and adapt our data, as long as you credit OpenStreetMap and its contributors. If you alter or build upon our data, you may distribute the result only under the same licence. The full legal code explains your rights and responsibilities.

Our documentation is licensed under the Creative Commons Attribution-ShareAlike 2.0 license (CC BY-SA 2.0).

How to credit OpenStreetMap

Where you use OpenStreetMap data, you are required to do the following two things:

- Provide credit to OpenStreetMap by displaying our copyright notice.
- Make clear that the data is available under the Open Database License.

For the copyright notice, we have different requirements on how this should be displayed, depending on how you are using our data. For example, different rules apply on how to show the copyright notice depending on whether you have created a browsable map, a printed map or a static image. Full details on the requirements can be found in the Attribution Guidelines.

To make clear that the data is available under the Open Database License, you may link to this copyright page. Alternatively, and as a requirement if you are distributing OSM in a data form, you can name and link directly to the license(s). In media where links are not possible (e.g. printed works), we suggest you direct your readers to openstreetmap.org (perhaps by expanding 'OpenStreetMap' to this full address) and to opendatacommons.org. In this example, the credit appears in the corner of the map.

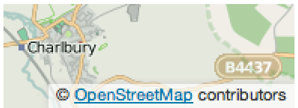

Finding out more

Read more about using our data, and how to credit us, at the OSMF Licence page.

Although OpenStreetMap is open data, we cannot provide a free-of-charge map API for third-parties. See our API Usage Policy, Tile Usage Policy and Nominatim Usage Policy.

Our contributors

Our contributors are thousands of individuals. We also include openly-licensed data from national mapping agencies and other sources, among them:

- **Austria**: Contains data from Stadt Wien (under CC BY), Land Vorarlberg and Land Tirol (under CC BY AT with amendments).
- **Australia**: Incorporates or developed using Administrative Boundaries © Geoscape Australia licensed by the Commonwealth of Australia under Creative Commons Attribution 4.0 International licence (CC BY 4.0).
- **Canada**: Contains data from GeoBase®, GeoGratis (© Department of Natural Resources Canada), CanVec (© Department of Natural Resources Canada), and StatCan (Geography Division, Statistics Canada).
- **Finland**: Contains data from the National Land Survey of Finland's Topographic Database and other datasets, under the NLSFI License.
- **France**: Contains data sourced from Direction Générale des Impôts.
- **Netherlands**: Contains © AND data, 2007 (www.and.com)
- **New Zealand**: Contains data sourced from the LINZ Data Service and licensed for reuse under CC BY 4.0.
- **Serbia**: Contains data from the Serbian Geodetic Authority and National Open Data Portal (public information of Serbia), 2018.
- **Slovenia**: Contains data from the Surveying and Mapping Authority and Ministry of Agriculture, Forestry and Food (public information of Slovenia).
- **Spain**: Contains data sourced from the Spanish National Geographic Institute (IGN) and National Cartographic System (SCNE) licensed for reuse under CC BY 4.0.
- **South Africa**: Contains data sourced from Chief Directorate: National Geo-Spatial Information, State copyright reserved.
- **United Kingdom**: Contains Ordnance Survey data © Crown copyright and database right 2010-2023.

For further details of these, and other sources that have been used to help improve OpenStreetMap, please see the Contributors page on the OpenStreetMap Wiki.

Inclusion of data in OpenStreetMap does not imply that the original data provider endorses OpenStreetMap, provides any warranty, or accepts any liability.

Copyright infringement

OSM contributors are reminded never to add data from any copyrighted sources (e.g. Google Maps or printed maps) without explicit permission from the copyright holders.

If you believe that copyrighted material has been inappropriately added to the OpenStreetMap database or this site, please refer to our takedown procedure or file directly at our on-line filing page.

Trademarks

OpenStreetMap, the magnifying glass logo and State of the Map are registered trademarks of the OpenStreetMap Foundation. If you have questions about your use of the marks, please see our Trademark Policy.

This page is available in the following languages:

Creative Commons License Deed

Attribution-ShareAlike 2.0 Generic (CC BY-SA 2.0)

This is a human-readable summary of (and not a substitute for) the license.

You are free to:

Share — copy and redistribute the material in any medium or format

Adapt — remix, transform, and build upon the material

for any purpose, even commercially.

The licensor cannot revoke these freedoms as long as you follow the license terms.

Under the following terms:

Attribution — You must give appropriate credit, provide a link to the license, and indicate if changes were made. You may do so in any reasonable manner, but not in any way that suggests the licensor endorses you or your use.

ShareAlike — If you remix, transform, or build upon the material, you must distribute your contributions under the same license as the original.

No additional restrictions — You may not apply legal terms or technological measures that legally restrict others from doing anything the license permits.

Notices:

You do not have to comply with the license for elements of the material in the public domain or where your use is permitted by an applicable exception or limitation.

No warranties are given. The license may not give you all of the permissions necessary for your intended use. For example, other rights such as publicity, privacy, or moral rights may limit how you use the material.

A new version of this license is available. You should use it for new works, and you may want to relicense existing works under it. No works are *automatically* put under the new license, however.
